# Non-Surgical Pneumoperitoneum After Fenestrated Endovascular Aortic Repair- Case Report

**DOI:** 10.1177/15385744261428751

**Published:** 2026-02-24

**Authors:** George Apostolidis, Giuseppe Panuccio, Petroula Nana, José I. Torrealba, Daour Yousef al Sarhan, Tilo Kölbel

**Affiliations:** 1 German Aortic Centre, Department of Vascular Medicine, University Heart and Vascular Centre UKE Hamburg, Hamburg, Germany

**Keywords:** pneumoperitoneum, fEVAR, endovascular aneurysm repair, mechanical ventilation, diaphragmatic hernia

## Abstract

**Objective:**

Post-operative pneumoperitoneum is mainly related to gastrointestinal perforation, although non-surgical pneumoperitoneum may also be present, with mechanical ventilation being the leading cause. Herein, we report a case of non-surgical pneumoperitoneum after percutaneous fenestrated endovascular aortic repair (fEVAR).

**Case report:**

A 79-year-old female presented with a 58 mm asymptomatic juxtarenal abdominal aortic aneurysm. The preoperative computed tomography angiography (CTA) detected also a diaphragmatic hernia. According to the latest guidelines, an indication for fEVAR was set. The successful implantation of a four-fenestrated custom-made endograft was performed using bilateral percutaneous femoral access. Even though the immediate postoperative period was uneventful, the predischarge CTA revealed a high-volume pneumoperitoneum and pneumomediastinum. The patient developed mild tenderness of the lower abdomen during palpation, and a postoperative elevation of the C-reactive protein (CRP = 205 mg/L) was identified. After general surgery consultation, an exploratory laparoscopy with intraoperative gastro-duodenoscopy were performed, which revealed no evidence of gastrointestinal perforation. The patient was discharged in good general condition on the sixth postoperative day.

**Conclusion:**

Post-operative pneumoperitoneum may be related to mechanical ventilation due to alveolar injury after fEVAR. Laboratory and imaging findings should be judged in the influence of clinical image. An initial watch and wait approach may be justified.

## Introduction

The presence of free air in the peritoneal cavity is mostly indicative of gastrointestinal perforation necessitating urgent surgical management.^1^ However, several other causes and various mechanisms, explaining free intra-abdominal air have been identified including abdominal, thoracic and gynecologic pathologies or interventions, with the most prevalent ones being iatrogenic. Pneumoperitoneum may be the result of retained post-operative air, peritoneal dialysis, gastrointestinal endoscopy, while other usual causes are mechanical ventilation, cardiopulmonary resuscitation and pneumothorax.^1,2^ Current evidence on pneumoperitoneum, not requiring surgical management, consists of case reports or case series.^2^ Non-surgical pneumoperitoneum after endovascular procedures has not been reported so far.

The aim of this case report is to present a patient who developed spontaneous pneumoperitoneum after percutaneous fenestrated endovascular aortic repair (fEVAR).

## Report

A 79-year-old female patient presented with an asymptomatic 58 mm juxtarenal abdominal aortic aneurysm. The patient had a history of hypertension, mild aortic valve stenosis, chronic kidney disease (stage IIIb) related to an atrophic left kidney and coronary artery disease, with prior myocardial infarction managed with percutaneous coronary intervention. In addition, a diaphragmatic hernia was detected in the pre-operative computed tomography angiography (CTA). The patient reported no related symptoms and had no history of prior surgical procedures. A written consent has been assigned by the patient, confirming the agreement to publication of the case details and images. Due to the anonymized data, no Ethics Committee approval was needed according to the current state law.

**Figure 1.**
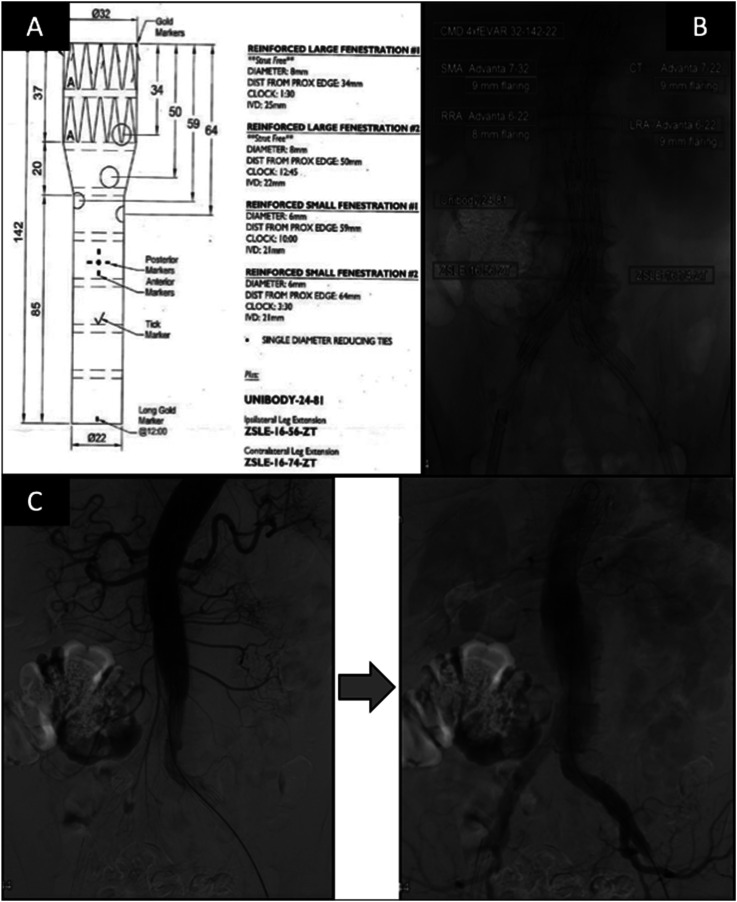
*Panel A:* Image of custom-made fenestrated graft patient-specific design. *Panel B:* Image of intraoperative angiography with information on specific stent grafts used in the procedure. *Panel C:* Successive images from completion angiography revealing a late endoleak near aortic bifurcation, probably type IIIa between main device and bifurcation device

According to the latest guidelines on complex abdominal aortic aneurysm management, an indication for repair was set.^3^ Due to the patient’s high-risk profile, endovascular management using a custom-made fenestrated device (Figure 1A) was decided.

### Procedure

The patient was operated in supine position under general anesthesia in a hybrid operating theatre under fusion guidance. Bilateral percutaneous femoral access was performed under ultrasonographic guidance, and a Manta closure system (Teleflex, Wayne, PA, USA) was deployed. Systemic heparinization with a target activated clotting time 250-300s was achieved. The main fenestrated device was introduced from the right common femoral artery (CFA) and deployed according to fusion markers. Through the left CFA access, a 22Fr sheath (XLSW, Cook Medical, Bloomington, IN, USA) was advanced into the fenestrated device and the target vessels were sequentially catheterized using a hydrophilic .035” guidewire, a 65 cm 5Fr Berenstein catheter and 6 or 7 Fr Flexor Ansel sheaths. After release of the diameter reducing ties and complete deployment of the device, all target vessels were bridged using balloon expandable covered stents (Advanta V12, Getinge/Atrium Medical Corporation, Merrimack, NH in all target vessels; celiac trunk: Advanta V12 7 × 22, superior mesenteric artery: Advanta V12 7 × 32, right renal artery: Advanta V12 6 × 22 and left renal artery: Advanta V12 6 × 22). The bifurcated device (Figure 1B) was then advanced and deployed, followed by iliac extensions (Figure 1B). The completion angiography revealed a type IIIa endoleak at the level of the iliac extensions and unimpaired flow to all target vessels (Figure 1C). The wire and sheaths were withdrawn from both groins and the closure system was uneventfully applied.

### Postoperative Course

The immediate postoperative period, including an initial intensive care unit (ICU) stay of 2 days, was uneventful. The clinical examination revealed no abnormal findings. The postoperative blood tests only showed an elevated CRP value of 205 mg/L. Leukocyte count and type remained normal. The rest of the laboratory findings were unremarkable.

**Figure 2.**
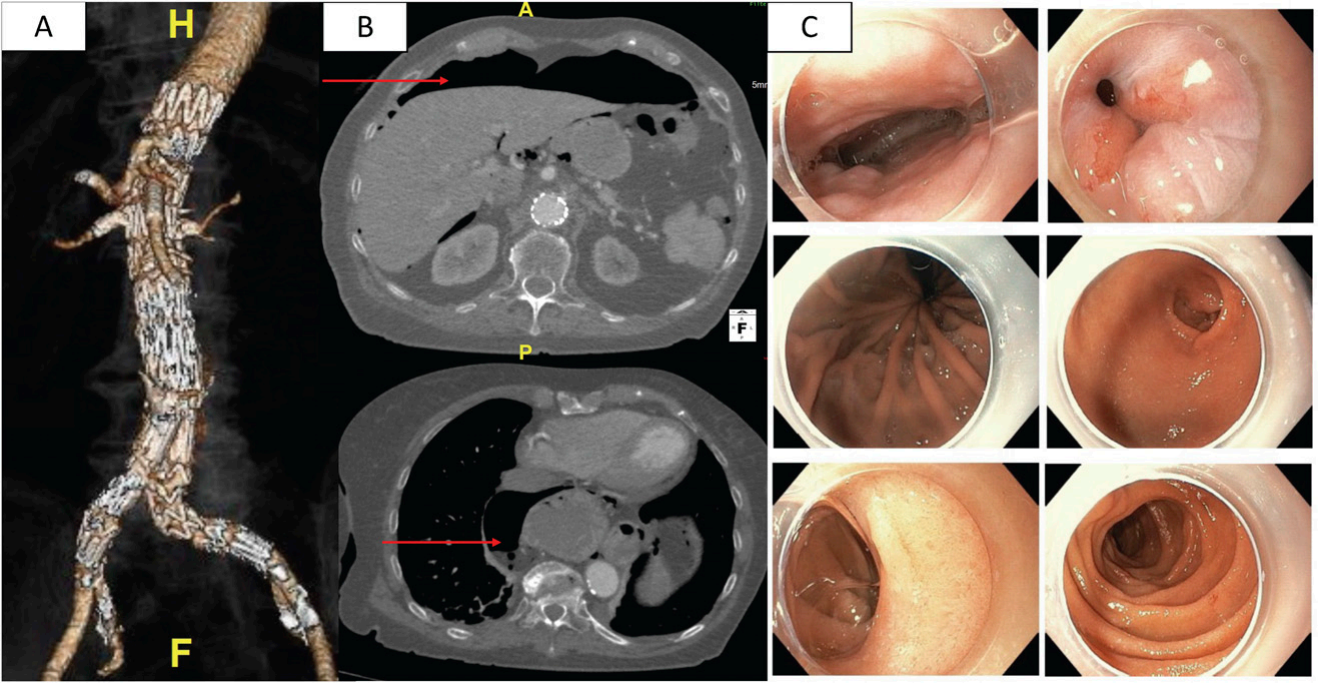
*Panel A:* 3D reconstruction of post-op CTA images, were no endoleak and a successful target vessel patency was revealed. *Panel B:* Presence of free air in peritoneum and in mediastinum (red arrows). *Panel C:* Images of intraoperative upper gastrointestinal endoscopy, confirming absence of any perforation sites

On the fourth postoperative day, a routine predischarge CTA revealed a complete aneurysm sac exclusion with no evidence of endoleak and preserved target vessel patency (Figure 2A). Surprisingly, a large volume of free air was detected in the peritoneal cavity, along with a concurrent pneumomediastinum connected through the large diaphragmatic hernia (Figure 2B). At this time, the patient exhibited a mild lower abdomen tenderness. No signs of hemodynamic instability were present.

Despite patient’s vague clinical presentation, the alarming imaging findings prompted a general surgery consultation. Based on the evaluation of imaging, laboratory and clinical findings, an immediate exploratory laparoscopy was performed, to rule out a gastrointestinal perforation. The laparoscopy revealed no evidence of perforation, and an intraoperative upper gastrointestinal endoscopy confirmed this finding, with a hiatal hernia being the only pathological condition observed (Figure 2C).

After the laparoscopy, the patient remained asymptomatic and in good general condition. No further adverse events were recorded. The patient was discharged at home on the sixth postoperative day. An antibiotic regimen was prescribed following the general surgeons’ instructions, along with dual antiplatelet treatment for 6 months.

## Discussion

The patient was managed surgically after the discovery of pneumoperitoneum in the predischarge CTA, despite that the clinical examination did not indicate an acute abdomen. The post-operative CTA control during the hospitalization instead of a later outpatient follow-up, may have contributed to the decision for interventional management. However, the timing of the post-operative CTA is driven by the center’s specific circumstances, serving a wider geographic area and the need to confirm the technical success of the procedure, avoiding early complications that would hamper patients’ further recovery or set the indication for reintervention (eg, need for relining in case of bridging stent stenosis or kinking). Imaging findings, along with the elevated CRP values, prompted the general surgeons’ decision to perform an exploratory laparoscopy. The simultaneous presence of free air in both the mediastinal and peritoneal cavities, along with the establishment of communication between those cavities through the diaphragmatic hernia and the lack of other pathology led to the suspicion of mechanical ventilation-related free intra-abdominal air.^4^ The proportion of surgically managed non-surgical cases of pneumoperitoneum ranges between 25-40%, according to the existing literature.^1,4^

Postoperative residual air presence in the peritoneal cavity after abdominal surgery- either open or laparoscopic- is among the main causes of non-surgical pneumoperitoneum.^1^ However, endovascular procedures have no obvious mechanism of intraperitoneal air accumulation, and no such case has been reported in the literature. The presence of intra-aortic and periaortic air in significantly small quantities has been described as a common postoperative finding, being acceptable during the immediate postoperative period, but related to graft infection when present at later stages.^5,6^

The absence of pathologic findings during laparoscopy and upper gastrointestinal endoscopy led us to consider mechanical ventilation as the most likely cause of the pneumoperitoneum in this patient. Mechanical ventilation has been proposed as one of the most prevalent causes of thorax-derived pneumoperitoneum, with an incidence up to 7% in ICU patients.^4^ The most comprehensive literature review revealed the simultaneous presence of pneumomediastinum or pneumothorax with pneumoperitoneum in all cases.^4^ From a pathophysiological perspective, ventilation induced lung injury, in connection with alveolar atelectasis, pressure changes or a ventilator-related inflammatory process, may lead to alveolar rupture.^7^ Bronchovascular sheaths containing bronchial and vascular branches serve as conduits through which air can travel to the mediastinum (Macklin effect).^8^ Further air migration to the peritoneal cavity is facilitated through the perivascular fasciae, as well as diaphragmatic hernias, which represent defects of the border between the mediastinum and peritoneal cavity.^9^ This mechanism appears to explain the presence of pneumoperitoneum in the present case. Ventilation related pneumoperitoneum is not always associated with concurrent pneumothorax; as according to the Macklin effect, when free air navigates, it prefers the path of the lower resistance, leading to the mediastinum. Access to the pleural space is not the “preferable” path due to its higher resistance. Vice versa, a simple-not tension-pneumothorax will not result in air migration to the pneumoperitoneum as intra-abdominal pressure normally exceeds intrathoracic pressure.^4,10^

The existence of non-surgical pneumoperitoneum has not been widely recognized by vascular surgeons. Greater awareness may increase clinical suspicion and help avoid unnecessary interventions. Existing diagnostic algorithms involve clinical examination, laboratory findings, alongside imaging results and typically suggest an initial watchful waiting approach, unless strong evidence warrants exploratory abdominal surgery.^2,11^ Pneumoperitoneum can present in a wide range of clinical scenarios, including both post-operative and non-postoperative setting.^2,11^ Clinical assessment- particularly evaluation of the hemodynamic stability and physical examination- should always form the foundation of diagnostic and therapeutic decision-making.^2^ This is especially important in cases of pneumoperitoneum following abdominal surgery.^11^ Concurrent imaging findings- such as presence of air in the pleural or mediastinal cavities, portal venous gas, intraluminal bowel air or free intra-abdominal fluid- can further guide diagnosis and management.^2,12^ Laboratory results, particularly inflammatory markers, may contribute to a more comprehensive assessment of the patient’s condition; however, they can be misleading in the postoperative setting.^2^

Surgical management in the absence of peritonitis has been shown to increase the perioperative morbidity and lead to a higher proportion of non-home discharge.^13^ However, even when an exploratory procedure is decided, laparoscopy instead of laparotomy may alleviate burden on patient’s general condition and facilitate the postoperative recovery, especially in case of hemodynamically stable patients with inconclusive findings. Cutting edge surgical modalities, like robotic assistance could further improve the available minimally invasive techniques, in terms of visualization, depth perception and precision, provided that they become widely available in the future. Further experience and studies are needed to evaluate the role of robotics in diagnostic laparoscopy.^11^

This case represents a rather rare entity, characterized by findings typically considered to warrant urgent surgical management but ultimately requiring conservative treatment. The recognition of non-surgical causes of pneumoperitoneum, adherence to the appropriate diagnostic and management algorithms and use of minimally invasive techniques are the key to a less invasive approach of these patients. Until further literature becomes available on this rare topic, awareness of non-surgical causes of pneumoperitoneum and management based on patients’ clinical status remain essential.

## Conclusion

Pneumoperitoneum after an uneventful percutaneous fEVAR may be related to alveolar injury caused by mechanical ventilation. Laboratory and imaging findings should be judged under the spectrum of the clinical findings and an initial watch and wait approach may be justified.
